# Correlations of mucin 5B gene polymorphisms and expression levels with the risk of onset of coal workers’ pneumoconiosis

**DOI:** 10.1097/MD.0000000000041088

**Published:** 2024-12-27

**Authors:** Li Yang, Hao Deng, Hongyun Chen, Mali Wu, Jun Li, Tao Zhang

**Affiliations:** a School of Public Health, Key Laboratory of Environmental Pollution Monitoring and Disease Control, Ministry of Education, Guizhou Medical University, Guiyang, China; b Guiyang Public Health Clinical Center, Guiyang, China; c Morphological Laboratory of Basic Medical College of Guizhou University of Traditional Chinese Medicine, Guiyang, China.

**Keywords:** coal workers’ pneumoconiosis, early diagnosis, gene, mucin 5B (MUC5B), pneumoconiosis, single-nucleotide polymorphism

## Abstract

This study investigates the correlations of mucin 5B (MUC5B) *rs2672794*, *rs2075854*, and *rs868903* polymorphisms and MUC5B expression level with the risk of the onset of coal workers’ pneumoconiosis (CWP). Overall, 506 Han Chinese men were included in this study. Among them, 143 were healthy individuals, 132 were dust-exposed workers who underwent health monitoring periodically, and 231 were patients with CWP. The participants were categorized into the following groups based on health status: healthy, exposure, CWP stage I, and CWP stage II groups. Genotyping was performed using the MassARRAY platform, and gene expression levels were measured via real-time fluorescence quantitative polymerase chain reaction. Subsequently, the correlations of 3 single-nucleotide polymorphisms (*rs2672794*, *rs2075854*, and *rs868903*) and MUC5B gene expression with the risk of CWP onset were analyzed. Distributions of the *rs2672794* (*P* = .82) and *rs2075854* (*P* = .72) genotypes were not significantly different among the various groups. Frequencies of the CC and CT genotypes of single-nucleotide polymorphism *rs868903* and the C allele of MUC5B were higher in patients with CWP than in the healthy group (*P* = .04). The MUC5B expression level of patients with CWP was significantly lower than those of the exposure and healthy groups (*P* < .001). Receiver operating characteristic curve analysis revealed that MUC5B in blood cells was a sensitive biomarker for CWP diagnosis. Significant differences were observed in MUC5B gene expression levels among different genotypes of *rs868903* (*P* < .001), with individuals carrying the CC and CT genotypes exhibiting lower MUC5B gene expression levels than those with the TT genotype. The *rs868903* polymorphism in the MUC5B gene could be associated with the susceptibility to CWP, and early monitoring would aid in identifying individuals at high risk. MUC5B might serve as a valuable early screening biomarker for CWP.

## 1. Introduction

Coal workers’ pneumoconiosis (CWP), also known as “black lung disease,” is a form of interstitial pulmonary fibrosis. The disease primarily affects coal miners and is caused by long-term inhalation of coal dust and its mixtures.^[1,2]^ CWP is one of the most severe occupational diseases in China. Currently, no specific indicators exist for the early warning and diagnosis of the disease.^[3]^ Its identification is mainly dependent on imaging changes; however, diagnosis based on such changes is limited. Detecting definite and visible pulmonary fibrotic lesions via imaging changes indicates severe impairment in the respiratory function and work capacity of the affected worker.^[4]^ Therefore, screening of biomarkers for early warning and diagnosis is crucial for CWP.

The pathogenesis of CWP remains unclear. Existing research has suggested that repeated stimulation of pulmonary tissue by coal dust particles generates severe inflammatory responses. In turn, such a pro-inflammatory environment triggers excessive proliferation of fibroblasts within the pulmonary interstitium, causing an overaccumulation of collagen in the extracellular matrix and ultimately resulting in diffuse pulmonary fibrosis.^[5]^ Mucin 5B (MUC5B), a representative secretory mucin in mammalian airways,^[6,7]^ has been shown to control inflammation and maintain immune stability. A study reported that MUC5B-deficient mice experienced severe impairment of macrophage phagocytic function, leading to a 93% reduction in the production of the anti-inflammatory cytokine interleukin-23.^[8]^ In a mouse model of chronic obstructive pulmonary disease, MUC5B knockout mice exhibited a significant increase in macrophage-associated inflammatory cytokines interleukin-6 and tumor necrosis factor-α, indicating that MUC5B can reduce pulmonary inflammation through the regulation of macrophage function.^[9]^ Notably, pneumoconiosis may be caused by interactions between genes and the environment, thus its association with individual genetic susceptibility.^[10,11]^ Recently, studies have been conducted on the correlations of single-nucleotide polymorphisms (SNPs) of various genes, including *LRBA*,^[12]^ *HMGB1*,^[13]^ and *VDR*,^[14]^ with the onset of pneumoconiosis. Moreover, a population study by Hunninghake et al^[15]^ revealed that the odds of imaging evidence of pulmonary fibrosis were 6.3× greater in individuals carrying MUC5B promoter polymorphisms. In addition, the expression level of MUC5B changes with the development of conditions, including chronic obstructive pulmonary disease, cystic fibrosis, and idiopathic pulmonary fibrosis.^[16–18]^ A genome-wide association study showed that SNP *rs868903* of the MUC5B promoter was strongly associated with pulmonary fibrosis.^[19]^ However, it remains unclear whether MUC5B is associated with the risk of onset of CWP. Therefore, this study aimed to investigate the correlations between MUC5B polymorphisms and expression level and the risk of onset of CWP.

## 2. dMaterials and methods

### 2.1. Study participants

We selected 143 healthy individuals who underwent pre-employment physical examinations, 132 coal workers who underwent on-the-job physical examinations, and 231 patients with CWP diagnosed with occupational diseases at Guiyang Public Health Treatment Center. All participants were Han Chinese men. Those with other pulmonary diseases (e.g., tuberculosis and lung cancer) or autoimmune diseases were excluded. The participants were grouped as follows: healthy individuals were assigned to the healthy group, coal miners were assigned to the exposure group, and patients with CWP were further categorized into CWP stage I and CWP stage II groups. A team of occupational disease diagnosis experts diagnosed all patients in accordance with the Chinese National Standard GBZ70-2015 (Diagnosis of Occupational Pneumoconiosis). Demographic information such as age, years of work experience, smoking history, and drinking history was collected through face-to-face interviews using a questionnaire survey. All participants signed a written informed consent form before the start of the study. In addition, the Ethics Committee of the Guiyang Public Health Clinical Center approved this study (No. 201920).

### 2.2. MUC5B SNP genotyping assay

By searching the PubMed and NCBI databases and screening functional sites based on the minor allele frequency of the SNP sites (minor allele frequency > .05), 3 SNPs (*rs2672794*, *rs2075854*, and *rs868903*) were ultimately selected. The genotype distributions of all SNPs of the study participants were in Hardy-Weinberg equilibrium (*P* > .05; Table 1). Genomic DNA was extracted using the TIANamp Blood DNA Kit (Tiangen Biochemical Technology Co., Ltd.) and stored in a −80 °C freezer before further use. Specific primers were designed for polymerase chain reaction (PCR) amplification. Following PCR, the PCR products were analyzed, and genotyping was performed using a MassARRAY mass spectrometer (Agena Bioscience, Inc). Raw data and genotyping results were analyzed using Typer 4.0 software (Agena Bioscience, Inc.).

**Table 1:** HWE testing of mucin 5B *rs2672794*, *rs2075854*, and *rs868903*.

rs No.	Location	Base	MAF*	HWE†
*rs2672794*	Intron	T > C	.303	.99
*rs2075854*	Intron	A > G	.395	.94
*rs868903*	Promoter	T > C	.454	>.99

HWE = Hardy-Weinberg equilibrium, MAF = minor allele frequency.

* MAF *P* values in the healthy group.

† HWE *P* values in the healthy group.

### 2.3. Gene expression

Total RNA was extracted from the blood samples of the participants using the EZ-press Whole Blood RNA Purification Kit (Shanghai Yishan Biotechnology Co., Ltd.) and reverse transcribed into 10 μL of cDNA via reverse-transcription PCR on a PCR system (Bio-Rad). β-actin was selected as the housekeeping gene for real-time fluorescence quantitative PCR to measure the gene expression levels of MUC5B. The reaction system had a total volume of 20 μL, comprising 10 μL of 2×SYBR Green Mix (Shanghai Yishan Biotechnology Co., Ltd.), 2 μL of cDNA template, 0.4 μL of both forward and reverse primers (Shanghai Sangong Bioengineering Co.), and 7.2 μL of double distilled water. The reaction conditions were set as follows: initial denaturation at 95 °C for 3 minutes, followed by 40 cycles of denaturation at 95 °C for 15 seconds, annealing for 30 seconds, and extension at 72 °C for 60 seconds. The primer sequences for MUC5B are given as follows: forward, 5’-TCCCACTATTCCACCTTTGACG-3’; reverse, 5’-CCAGGTAGAGGCTGAGATTCCC-3’. For β-actin, the sequences are given as follows: forward, 5’-CTAAGTCATAGTCCGCCTAGAAGCA-3’; reverse, 5’-TGGCACCCAGCACAATGAA-3’.

### 2.4. Statistical analysis

Data were statistically analyzed using IBM SPSS Statistics for Windows, version 23.0 (IBM Corporation, Armonk, NY). Normally distributed quantitative data were expressed as X ± S and compared using a 1-way analysis of variance. Nonnormally distributed quantitative data were expressed as median and quartiles (*P*_25_ and *P*_75_) and compared using the Kruskal-Wallis *H* test. Frequencies of count data were compared using the χ^2^ test for R × C contingency tables. The Hardy-Weinberg equilibrium testing for the assessment of population representativeness of the study sample was performed using the χ^2^ test. Differences were considered statistically different when *P* < .05.

## 3. Results

### 3.1. Basic data of study participants

Overall, 143, 132, 121, and 110 participants in the healthy, exposure, CWP stage I, and CWP stage II groups, respectively, were included in this study. Table 2 presents the demographic data of the study participants. Participants in the exposure and CWP groups were older and had longer years of work experience than those in the healthy group, with the differences being statistically significant (*P* < .001). Differences in smoking history (*P* = .08) and alcohol consumption status (*P* = .23) among the participants were not statistically significant.

**Table 2:** Demographic data of the study participants.

Variables	Healthy groups (n = 143)	Exposure group (n = 132)	CWP stage I (n = 121)	CWP stage II (n = 110)	*P* value
Age, yr; M (*P*_25_, *P*_75_)	27 (22, 30)	49 (46, 52)	53 (48.5, 55)	53 (50, 57)	<.001
Exposure years, M (*P*_25_, *P*_75_)	0	15 (12, 25.5)	14.5 (10, 22.75)	14.5 (10.5, 19.25)	<.001
Smoking status					.08
Never	64 (44.8)	41 (31.8)	47 (39.2)	43 (43.0)	
Ever	16 (11.2)	11 (8.5)	10 (8.3)	15 (15.0)	
Current	63 (44.1)	77 (59.7)	63 (52.5)	42 (42.0)	
Alcohol consumption status					.23
Never	77 (53.8)	55 (42.6)	58 (48.3)	48 (48.0)	
Former	61 (42.7)	66 (51.2)	51 (942.5)	42 (42.0)	
Current	5 (3.5)	8 (6.2)	11 (9.2)	10 (10.0)	

CWP = coal workers’ pneumoconiosis, M = median.

### 3.2. MUC5B genotyping

Table 3 shows the frequencies of genotypes and alleles of the 3 SNPs in the MUC5B gene of all participants. Distributions of the *rs2672794* (*P* > .82) and *rs2075854* (*P* > .72) genotypes were not significantly different among the various groups. Compared with the healthy group, the exposure, CWP stage I, and CWP stage II groups showed a decrease in the wild-type TT and an increase in the mutant types CC and CT for MUC5B *rs868903*, with the differences being statistically significant (*P* = .04). The frequency of the C allele was higher in the exposure and CWP groups than in the healthy group, with the difference being statistically significant (*P* = .04).

**Table 3:** Genotype distributions of mucin 5B in different groups.

Variables	Healthy groups		Exposure group		CWP stage I		CWP stage II		*P* value
	n	%	n	%	n	%	n	%	
*rs2672794*									.82
CC	15	10.7	21	15.9	19	16.0	13	12.3	
CT	67	47.9	56	42.4	53	44.5	52	49.1	
TT	58	41.4	55	41.7	47	39.5	41	38.7	
C allele	97	34.6	98	37.1	91	38.2	78	36.8	.86
T allele	183	65.4	166	62.9	147	61.8	134	63.2	
*rs2075854*									.72
AA	33	23.1	30	22.7	26	21.5	29	26.4	
AG	64	44.8	63	47.7	65	53.7	47	42.7	
GG	46	32.2	39	29.6	30	24.8	34	30.9	
A allele	130	45.4	123	46.6	117	48.3	105	47.7	.92
G allele	156	54.6	141	53.4	125	51.7	115	52.3	
*rs868903*									.04
TT	42	29.4	20	15.2	23	19.0	16	14.5	
CT	63	44.0	65	49.2	61	50.4	61	55.5	
CC	38	26.6	47	35.6	37	30.6	33	30.0	
T allele	147	51.4	105	39.8	107	44.2	93	42.3	.04
C allele	139	48.6	159	60.2	135	55.8	127	57.7	

CWP = coal workers’ pneumoconiosis.

### 3.3. MUC5B expression and receiver operating characteristic curve analysis

**Figure 1.**
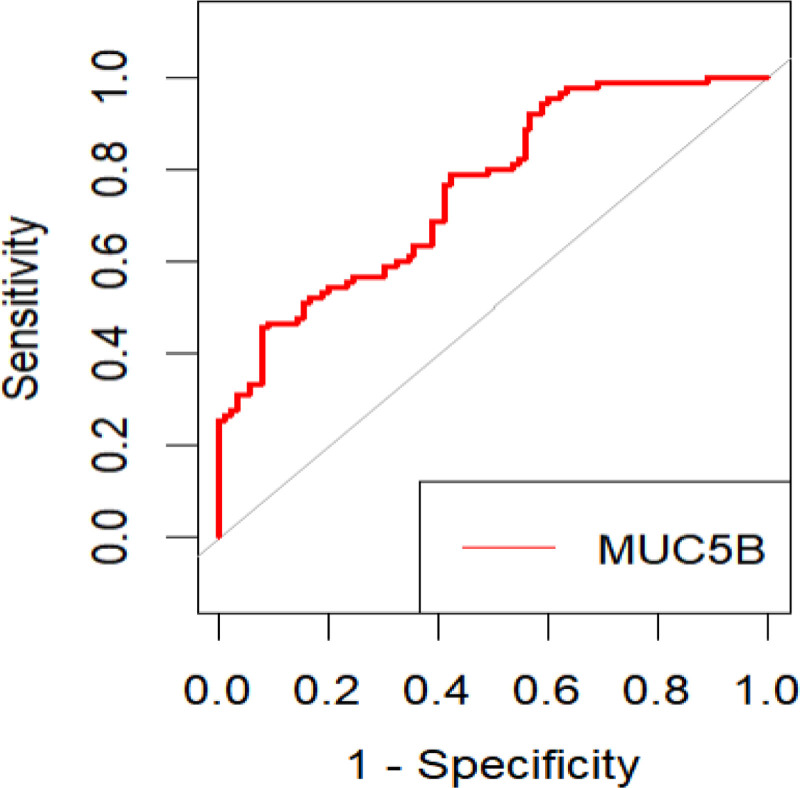
Results from mucin 5B (MUC5B) receiver operating characteristic curve analysis.

The expression levels of MUC5B in the CWP stage I and CWP stage II groups were decreased compared with those in the healthy and exposure groups, with the differences being statistically significant (*P* < .001). No statistically significant difference was found in expression between the healthy and exposure groups, as shown in Table 4. The receiver operating characteristic (ROC) curve analysis was performed with a confirmed diagnosis of CWP set as the gold standard and the MUC5B expression level of the participants set as the test variable. Results indicated that the area under the ROC curve was 0.759, which was greater than 0.700 and within the range of 0.700 to 0.900. Sensitivity and specificity were 92.2% and 45.6%, respectively. Therefore, MUC5B possesses a moderate diagnostic value and may serve as an auxiliary diagnostic indicator for CWP, with a diagnostic cutoff value of 0.557 (Table 5; Fig. 1).

**Table 4:** MUC5B gene expression levels.

Groups	n	MUC5B, M (*P*_25_, *P*_75_)	*H*	*P*
Healthy group	45	1.220 (.854, 2.004)	39.788	<.001
Exposure group	45	1.125 (.734, 1.657)		
CWP stage I	45	.849 (.351, 1.059)		
CWP stage II	45	.492 (.222, 1.019)		

CWP = coal workers’ pneumoconiosis, M = median, MUC5B = mucin 5B.

**Table 5:** Receiver operating characteristic curve of mucin 5B.

AUC	Standard error	95% CI	Cutoff	Sensitivity, %	Specificity, %	*P*
0.759	0.035	0.691–0.827	0.557	92.2	45.6	<.001

AUC = area under the receiver operating characteristic curve, CI = confidence interval.

### 3.4. Analysis of the association between different genotypes of rs868903 and MUC5B expression levels

Grouping individuals based on different genotypes of rs868903 and analyzing the expression level of the MUC5B gene revealed that the expression level of the MUC5B gene in carriers of the CC and CT genotypes was decreased compared with the TT genotype, and the difference was statistically significant (*P* < .001), as presented in Table 6.

**Table 6:** Comparative analysis of mucin 5B expression across rs868903 genotypic variants.

*rs868903*	MUC5B, M (*P*_25_, *P*_75_)]	*P*
TT	1.096 (0.429, 1.655)	.041
CT	0.828 (0.410, 1.177)	
CC	1.041 (0.673, 1.361)	

M = median, MUC5B = mucin 5B.

## 4. Discussion

CWP is a chronic inflammatory pulmonary disease influenced by both environmental and genetic factors. Hou et al^[20]^ and Yuan et al^[21,22]^ have reported that individual genetic susceptibility plays a critical role in the onset and development of CWP. The correlations of MUC5B polymorphisms with the risk of onset of CWP have rarely been investigated. Polymorphism at the specific site *rs868903* of the MUC5B gene has been associated with honeycomb changes in the high-resolution computed tomography images and mortality rate of Chinese patients with idiopathic pulmonary fibrosis.^[23]^ In this study, we found that the frequencies of the *rs868903* CC and CT genotypes were significantly higher in the CWP population than in the healthy group, suggesting a potential association of these genotypes with CWP susceptibility. The proportion of individuals carrying the C allele was also higher in patients with CWP than in the healthy and exposure groups. This result further demonstrates that *rs868903* is a gene locus for CWP susceptibility, and the C allele may be a risk factor for the onset of CWP. Previous studies have revealed correlations between the MUC5B gene and various pulmonary diseases.^[24–28]^ The association between MUC5B and CWP observed in this study reveals that similar molecular mechanisms may also be present in CWP.

*rs868903* is located on the promoter region of the MUC5B gene, and its polymorphism may alter MUC5B expression by influencing the transcription levels of the gene. In this study, we also investigated the relationship between MUC5B gene expression level and the risk of CWP in a Chinese population. Our results revealed that the MUC5B mRNA expression level was closely associated with CWP. Compared with the healthy group, the exposure and CWP groups exhibited a decrease in the average expression level of MUC5B, with significant differences observed between the CWP group and the healthy and exposure groups. This suggests that the MUC5B expression level decreased with the onset and development of CWP, which is consistent with the findings of studies on certain airway-related diseases.^[29–31]^ Specifically, the MUC5B mRNA expression level was not significantly different between the healthy and exposure groups, which may be attributed to the physiological regulatory effects of the body during the early stage of dust exposure. Mucociliary clearance in the respiratory tract effectively eliminates inhaled coal dust particles through the mechanical transport function of cilia,^[32,33]^ thereby maintaining airway hygiene. Moreover, the active secretion of MUC5B during this process contributes to the formation of a strong protective barrier. However, continued inflammatory responses and fibrotic processes during the development of CWP may reduce MUC5B secretion. We further performed ROC curve analysis to determine whether MUC5B can serve as a possible auxiliary diagnostic biomarker for CWP. Results indicated that MUC5B was a relatively sensitive biomarker for CWP diagnosis in coal dust-exposed workers, suggesting that changes in MUC5B in the blood cells of workers should be closely monitored during the dust exposure period, and the cutoff value should be used as the diagnostic threshold.

Overall, the *rs868903* polymorphism may be associated with susceptibility to CWP. Therefore, detecting MUC5B gene polymorphisms may assist in the clinical screening of susceptible individuals for CWP. However, this study found no association between the *rs2075854* and *rs2672794* polymorphisms and CWP. The discovery of these genetic associations provides the potential for developing early detection methods. By detecting an individual’s MUC5B expression level or *rs868903* polymorphism, high-risk individuals who are susceptible to CWP can be identified. Our study results also suggest that a decrease in MUC5B expression level is associated with the occurrence and development of CWP, indicating that MUC5B may be a potential therapeutic target. Researchers can explore the development of drugs targeting MUC5B to restore its expression level, thereby slowing the progression of the disease. Notably, these findings of genetic associations require further validation and in-depth study. Genetic factors are only one aspect of the development of CWP, and environmental exposures and other nongenetic factors also play important roles. Therefore, prevention strategies and treatment approaches should consider genetic and environmental factors in combination with effective occupational health measures and medical interventions.

Due to regional, ethnic, and environmental differences that may affect the research results, there are certain limitations to the findings of this study. In future research, we need to further expand the sample size and include more coal worker pneumoconiosis patients, healthy populations, and dust-exposed workers from different geographical, gender, and ethnic backgrounds. At the same time, we need to increase the longitudinal follow-up of the study participants to provide stronger evidence for the prevention and early diagnosis of coal worker pneumoconiosis. In addition, efforts should be made to more accurately quantify coal dust exposure and adopt advanced statistical methods to address the limitations identified in our research.

## 5. Conclusion

This study provides a theoretical research basis for the screening of candidate biomarkers for the prevention and early warning of CWP. MUC5B *rs868903* polymorphism may be associated with CWP susceptibility. Testing for MUC5B gene polymorphisms may facilitate clinical screening in the CWP-susceptible population. Therefore, MUC5B may serve as an effective screening marker for early warning of CWP.

## Acknowledgments

The authors appreciate the contributions and support of all authors. They also thank all participants for providing blood samples.

## Author contributions

**Conceptualization:** Li Yang, Hao Deng

**Data curation:** Li Yang, Hao Deng, Hongyun Chen, Mali Wu

**Formal analysis:** Li Yang, Hao Deng

**Investigation:** Li Yang, Hao Deng

**Methodology:** Li Yang, Hao Deng

**Validation:** Li Yang, Hao Deng, Jun Li

**Writing – original draft:** Li Yang

**Writing – review & editing:** Hao Deng

**Supervision:** Jun Li

**Funding acquisition:** Tao Zhang

**Project administration:** Tao Zhang

**Resources:** Tao Zhang

**Software:** Tao Zhang
